# Aripiprazole Improves Associated Comorbid Conditions in Addition to Tics in Adult Patients with Gilles de la Tourette Syndrome

**DOI:** 10.3389/fnins.2016.00416

**Published:** 2016-09-12

**Authors:** Sarah Gerasch, Ahmad Seif Kanaan, Ewgeni Jakubovski, Kirsten R. Müller-Vahl

**Affiliations:** ^1^Clinic of Psychiatry, Socialpsychiatry and Psychotherapy, Hannover Medical SchoolHannover, Germany; ^2^Max Planck Institute for Human Cognitive and Brain SciencesLeipzig, Germany

**Keywords:** Tourette Syndrome, aripiprazole, OCD, depression, anxiety, ADHD, premonitory urge, quality of life

## Abstract

Gilles de la Tourette Syndrome (GTS) is characterized by motor and vocal tics, as well as associated comorbid conditions including obsessive-compulsive disorder (OCD), attention deficit/hyperactivity disorder (ADHD), depression, and anxiety which are present in a substantial number of patients. Although randomized controlled trials including a large number of patients are still missing, aripiprazole is currently considered as a first choice drug for the treatment of tics. The aim of this study was to further investigate efficacy and safety of aripiprazole in a group of drug-free, adult patients. Specifically, we investigated the influence of aripiprazole on tic severity, comorbidities, premonitory urge (PU), and quality of life (QoL). Moreover, we were interested in the factors that influence a patient's decision in electing for-or against- pharmacological treatment. In this prospective uncontrolled open-label study, we included 44 patients and used a number of rating scales to assess tic severity, PU, comorbidities, and QoL at baseline and during treatment with aripiprazole. Eighteen out of fortyfour patients decided for undergoing treatment for their tics with aripiprazole and completed follow-up assessments after 4–6 weeks. Our major findings were (1) aripiprazole resulted in significant reduction of tics, but did not affect PU; (2) aripiprazole significantly improved OCD and showed a trend toward improvement of other comorbidities including depression, anxiety, and ADHD; (3) neither severity of tics, nor PU or QoL influenced patients' decisions for or against treatment of tics with aripiprazole; instead patients with comorbid OCD tended to decide in favor of, while patients with comorbid ADHD tended to decide against tic treatment; (4) most frequently reported adverse effects were sleeping problems; (5) patients' QoL was mostly impaired by comorbid depression. Our results suggest that aripiprazole may improve associated comorbid conditions in addition to tics in patients with GTS. It can be hypothesized that these beneficial effects are related to aripiprazole's adaptive pharmacological profile, which exhibits an influence on the dopaminergic as well as a number of other neurotransmitter systems. For the first time, our data provide evidence that patients' decision making process for or against medical treatment is influenced by other factors than tic severity and QoL.

## Introduction

Gilles de la Tourette Syndrome (GTS) is a chronic neuropsychiatric disorder with childhood onset. GTS is characterized by multiple motor and one or more vocal tics (American Psychiatric Association, 2013). Tics are defined as rapid, non-rhythmic, involuntary movements or vocalizations that are misplaced in context and time (Jankovic, 1997). The majority of adult patients is able to suppress their tics voluntarily (Müller-Vahl et al., 2014) and most adults report premonitory urge (PU) sensations prior to their tics (Leckman et al., 1993). Contrary to the general belief and patients' reports, recent studies have been suggested that there is no direct relation between tic severity, PU, and tic suppression (Ganos et al., 2012; Müller-Vahl et al., 2014).

Today, it is well-known that 80–90% of patients with GTS in addition suffer from comorbid disorders such as obsessive-compulsive disorder (OCD), depression, anxiety, and attention deficit/hyperactivity disorder (ADHD) (Freeman et al., 2000; Leckman, 2002). At such, there is an ongoing debate concerning the number of valid sub-classifications within the GTS spectrum. For example, Robertson (2000) suggested the following classification: pure GTS (tics only), full-blown GTS (plus complex tics), and GTS plus (including comorbidities). More recent work has provided strong evidence that comorbid OCD, anxiety, and depression belong to the GTS spectrum, while comorbid ADHD should be classified as a separate problem (Lebowitz et al., 2012; Hirschtritt et al., 2015; Trillini and Müller-Vahl, 2015a). In adult patients with GTS, it has been demonstrated that health-related quality of life (QoL) is remarkably influenced by psychiatric comorbidities, in particular depression and OCD (Müller-Vahl et al., 2010; Jalenques et al., 2012).

Although varied therapeutic strategies currently being used to treat patients with GTS (e.g., behavioral therapy, pharmacotherapy, and deep brain stimulation), no currently available intervention has been shown to be able to effectively target the multiple symptoms associated with GTS. With respect to pharmacological treatment, current recommendations are based on a small number of controlled or uncontrolled studies, as large scale and longitudinal randomized controlled trials (RCTs) including a larger number of patients over a longer period of time have not been undertaken to date (Roessner et al., 2011; Weisman et al., 2014). Nevertheless, there is general agreement that substances that affect the dopaminergic system (antipsychotics) are most effective in the treatment of tics (Singer, 2010).

Since the discovery of its tic suppressive effects in the 1970s, haloperidol still stands as the sole formally approved medication for the treatment of GTS in most European countries. However, many clinicians no longer recommend it as haloperidol exhibits a significant adverse effect (AE) profile (Shapiro et al., 1973).

Although not licensed, other (second generation) antipsychotics such as risperidone, sulpiride, tiapride, and in particular aripiprazole are the most common medications used today for the treatment of tics, mainly as a result of their more favorable side effect profile. Within this class of drugs, aripiprazole has become the preferred antipsychotic in many centers for treating tics (Roessner et al., 2011; Hartmann and Worbe, 2013), since it is suggested that it causes less side effects (Wenzel et al., 2012) and is also effective in severely affected and otherwise treatment-refractory patients (Roessner et al., 2011).

Since 2004, various reports exploring the efficacy and safety of aripiprazole in the treatment of tics in GTS have been published. These include 28 case reports (Hounie et al., 2004; Dehning et al., 2005; Kastrup et al., 2005; Murphy et al., 2005, 2009; Padala et al., 2005; Bubl et al., 2006; Constant et al., 2006; Davies et al., 2006; Duane, 2006; Fountoulakis et al., 2006; Yoo et al., 2006, 2007; Ben Djebara et al., 2008; Budman et al., 2008; Findling et al., 2008; Seo et al., 2008; Winter et al., 2008; Ikenouchi-Sugita et al., 2009; Kawohl et al., 2009; Lai, 2009; Lyon et al., 2009; Cui et al., 2010; Frölich et al., 2010; Masi et al., 2012; Wenzel et al., 2012; Diomšina et al., 2015; Mazlum et al., 2015), 3 controlled trials (Gulisano et al., 2011; Yoo et al., 2013; Ghanizadeh and Haghighi, 2014), and 1 systematic review (Ghanizadeh, 2012). However, less than 40% of these reports have investigated the effects of aripiprazole on adult populations. In the two largest children only studies, aripiprazole was found to be superior to placebo (Yoo et al., 2013) and comparably effective as risperidone (Ghanizadeh and Haghighi, 2014) with respect to the improvement of tics and QoL. In general, aripiprazole was well tolerated. The most frequently reported AEs were drowsiness, increased appetite and weight gain. Based on these preliminary data, Ghanizadeh (2012) concluded in his systematic review that aripiprazole is effective in the treatment of tics in both adults and children with GTS with an adverse effect profile that seems to be safer than in other antipsychotic drugs. To the best of our knowledge, so far, no data are available on the effect of aripiprazole on PU.

Interestingly, in most of these studies effects of aripiprazole on tics have been investigated, but not on psychiatric comorbidities. Since aripiprazole influences the dopaminergic, and other neurotransmitter systems including the serotonergic, GABAergic, and glutamatergic systems (De Bartolomeis et al., 2015), it can be speculated that in addition to tics, it ameliorates comorbid symptoms including OCD, ADHD, depression, rage attacks, and anxiety. As a result in this work, we aimed at investigating the efficacy and safety of aripiprazole in a relatively large sample of unmedicated adult patients with GTS, specifically focusing on its effects on tics, QoL, PU, and psychiatric comorbidities (OCD, ADHD, depression, and anxiety). Since no other psychotropic drugs were allowed, influences from other drugs or interactions with other substances could be excluded. Our study design additionally allowed us to investigate difference in clinical features between patients who elected for (and against) treatment with aripiprazole.

We hypothesized that (1) aripiprazole improves both tics and behavioral problems in unmedicated adult patients with GTS and that (2) tic severity and QoL have the strongest influence on patients' decision for treatment with aripiprazole.

## Methods

This study has been performed as part of the EU-funded Marie Curie Initial Training Network (ITN) TS-EUROTRAIN (FP7-PEOPLE-2012-ITN, Grant Agr.No.316978). Patients were investigated at baseline and following treatment with aripiprazole using a variety of clinical tools. We decided to use both self- and expert ratings in order to obtain higher validity. Self-ratings solely based on patient's judgment. Expert-ratings were performed by a team of 2 psychologists and 1 physician either at the Hannover Medical School (MHH) or the Institute for Human Cognitive and Brain Sciences at the Max-Planck-Institute in Leipzig. In most cases, the respective patient was assessed at baseline and follow-up by the same rater. In addition, in all patients Magnetic Resonance Imaging (MRI) was performed (at the Institute for Human Cognitive and Brain Sciences at the Max-Planck-Institute, Leipzig) both at baseline and after treatment with aripiprazole. Neuroimaging results of this study will be published elsewhere.

### Subjects

In this study, 44 patients with GTS according to DSM-5 were included. Patients using any psychoactive substances underwent a 4-week washout period before participation. Exclusion criteria were age < 18 and >65 years, inability to lie still in the MRI, MRI contraindications, pregnancy, and breast-feeding. Patients were recruited from the Clinic of Psychiatry, Socialpsychiatry and Psychotherapy at the MHH and via newsletters, internet and the German Tourette advocacy groups between May 2014 and October 2015. Ethics approval was granted by the ethics committees both at the MHH and the University of Leipzig. All participants gave written informed consent before entering the study. Patients received a monetary compensation for study participation.

### Design

After baseline investigations, to all patients aripiprazole was offered for treatment of tics. Aripiprazole was gradually up-titrated every 3 days starting with 2.5 mg/day up to a maximum dose of 30 mg/day. Dosage was increased individually until the patient reached his/her individually tolerated maximum dose or best tic improvement defined on the basis of patient's judgment and investigator's assessments. Thus, no target dose was predefined and the final dose could range from 2.5 to 30 mg/day. Aripiprazole was administered once daily in the morning or in case of significant sedation, alternatively in the evening. In those patients, who decided for treatment with aripiprazole, investigations were performed again after 4–6 weeks treatment with aripiprazole. After study completion, patients could decide either to continue or to discontinue treatment.

### Clinical assessments

All patients underwent a neuropsychiatric interview and a comprehensive clinical assessment battery including measurements of tics, PU, QoL, and psychiatric comorbidities (OCD, ADHD, depression, anxiety, and autism).

#### Assessments for tics

1. Yale Global Tic Severity Scale (YGTSS) (Leckman et al., 1989): The YGTSS is a scale for the assessment of number, frequency, intensity, complexity, and interference of motor and vocal tics, and for the estimation of the global impairment; “total tic score” (TTS) (range, 0–50), divided in “motor tic score” (MT) (range, 0–25) and “vocal tic score” (VT) (range, 0–25), “overall impairment score” (range, 0–50), and “global score” (GS) (range, 0–100).
2. Modified Rush Video-Based Tic Scale (MRVS) (Goetz et al., 1999): The 10-min film protocol includes full frontal body (far) and head/shoulders only (near) views with and without an examiner in the room lasting 2.5 min each. For tic rating, only two (far and near body views) 1-min recordings with no examiner present were scored rating 5 domains: number of body areas involved (range, 0–4), motor tic frequency (range, 0–4), vocal tic frequency (range, 0–4), motor tic severity (range, 0–4), vocal tic severity (range, 0–4) resulting in the total score (range, 0–20).

#### Assessment for PU

Premonitory Urge for Tics Scale (PUTS) (Woods et al., 2005): The PUTS is a self-rating for PU (range 0–36); the higher the sum, the higher the PU.

#### Assessments for health-related QoL

The Gilles de la Tourette Syndrome Quality of Life Scale (GTS-QoL) and satisfaction-with-life visual analog scale (GTS-QoL-VAS) (Cavanna et al., 2008) were used to assess health-related QoL (range, 0–100, each): the higher the GTS-QoL score, the lower the patients' QoL; the higher the GTS-QoL-VAS, the higher the satisfaction-with-life.

#### Assessments for psychiatric symptoms

1. M.I.N.I. International Neuropsychiatric Interview 5.0 (Sheehan et al., 2006): The M.I.N.I. is an abbreviated version of the Structured Clinical Interview for DSM-IV (SCID) (Wittchen et al., 1997) based on ICD-10 classifications of mental and behavioral disorders. It is divided into 16 modules, each corresponding to a diagnostic category. At the end of each module, diagnostic box(es) allow the interviewer to specify whether diagnostic criteria for the respective clinically relevant disease are met (Goodman et al., 1989).
2. Montgomery Asberg Depression Scale (MADRS) (Montgomery and Asberg, 1979): MADRS is an examiner rating for the diagnosis and severity of depression (range, 0–60), 0–6 = normal/symptoms absent, 7–19 = mild depression, 20–34 = moderate depression, ≥35 = severe depression.
3. Beck Depression Inventory II (BDI-II) (Beck et al., 1996; Hautzinger et al., 2006): BDI-II is a self-report scale for depression (range, 0–63); 0–12 = no depressive symptoms, 13–19 = mild, 20–28 = medium, and ≥29 = severe depressive symptoms. Cut-off for clinically relevant depressive symptoms is ≥18.
4. Beck Anxiety Inventory (BAI) (Beck et al., 1988; Margraf and Ehlers, 2007): This self-rating was used to measure anxiety symptoms (range, 0–63), 0–7 = minimum level anxiety, 8–15 = mild, 16–25 = medium, and ≥26 = serious level anxiety. Cut-off for clinically relevant anxiety symptoms is ≥26.
5. Yale-Brown Obsessive Compulsive Scale (Y-BOCS) (Goodman et al., 1989): The Y-BOCS was used to assess obsessive-compulsive symptoms, obsessions (range, 0–24), cut-off for clinical relevant obsessions ≥10, compulsions (range, 0–24), cut-off for clinical relevant compulsions ≥10, for both (range, 0–48), cut-off ≥16.
6. Obsessive-Compulsive Inventory Revised (OCI-R) (Foa et al., 2009): in addition, we used the OCI-R for the measurement of OCD and obsessive-compulsive behavior (OCB). It is a self-rating-scale including a five-point Likert-type scale from 0 (“not at all”) to 4 (“extremely”). The scale consists of 6 three-item subscales (range, 0–12 each): washing (cut-off = 3), checking (cut-off = 5), ordering (cut-off = 7), obsessing (cut off = 5), hoarding (cut-off = 5) and mental neutralization (cut-off = 3).
7. DSM-IV symptom list for ADHD (Rösler et al., 2004): this retrospective questionnaire (range, 0–18) includes 2 domains; attention deficit disorder (cut-off ≥6) and hyperactivity disorder (cut-off ≥6).
8. Wender Utah Rating Scale (WURS-k) (Ward et al., 1993; Retz-Junginger et al., 2003): The WURS-k was used as a retrospective self-rating (range, 0–100), cut-off ≥30 suggests the diagnosis of ADHD.
9. Conners Adult ADHD Rating Scale (CAARS) (Christiansen et al., 2011): The CAARS was used to measure current symptoms (range, 0–198). Raw scores have to be converted into T-scores (cut-off ≥65) for each category: inattention/memory problems, hyperactivity/restlessness, impulsivity/emotional lability, problems with self-concept, DSM-IV inattentive symptoms, DSM-IV hyperactive-impulsive symptoms, DSM-IV ADHD symptoms total, and ADHD index.
10. Autism-Spectrum-Quotient (AQ) (Baron-Cohen et al., 2001): The AQ was used to measure autistic traits (range, 0–50), cut-off ≥32. The AQ cannot be used to make the diagnosis of an autism spectrum disorder.

#### Diagnoses of comorbidities

In order to (1) achieve a reliable diagnosis of psychiatric comorbidities, (2) reduce the rate of type-I and type-II errors, and (3) avoid a bias due to erroneous self-perception, we utilized a number of assessment tools, including both self-rating and expert-rating scales, to diagnose each condition. Accordingly, the particular diagnoses were made as follows:

Diagnosis of depression: For the diagnosis of depression, results of both M.I.N.I., MADRS, and BDI-II were taken into account as suggested earlier (Uher et al., 2008; Van Noorden et al., 2012; Van der Lem et al., 2015). Patients were diagnosed as depressive if they fulfilled (1) the M.I.N.I. category “major depressive episode current” and reach a BDI-II score ≥18 and/or a MADRS score ≥7 or (2) reached both a BDI-II score ≥18 and a MADRS score ≥7.

Diagnosis of anxiety disorder: For the diagnosis of anxiety disorder, results of both M.I.N.I. and BAI were taken into consideration (Phan et al., 2016). Anxiety disorder was diagnosed if the patients (1) fulfilled the category “panic disorder current,” “agoraphobia current,” “social phobia current,” and/or “generalized anxiety disorder current (GAD)” according to M.I.N.I. and reach a BAI score ≥8 or (2) reached a BAI score ≥26.

Diagnosis of OCD: 3 different rating scales were used to assess OCD in which the patient had to fulfill the M.I.N.I. category “OCD current” and the respective cut-off values of the Y-BOCS and/or the OCI-R (in at least one subscale).

Diagnosis of ADHD: As suggested elsewhere, the diagnosis of current ADHD was made based on results obtained from DSM-IV symptom list, WURS-k, and CAARS (Taylor et al., 2011; Smyth and Meier, 2016). Patients were diagnosed with ADHD if they satisfied respective cut-off values of WURS-k or DSM-IV symptom list and of ≥4/8 CAARS categories.

#### GTS subgroup classification and comorbidity score

Depending on the presence of comorbid diagnoses as defined above, the following subgroup classifications were used for further analysis: “GTS only” (without comorbid OCD, ADHD, depression, and anxiety disorder) and “GTS plus” (with ≥1 of above mentioned comorbidities). In addition, we defined the following sub-classifications depending on the kind of the psychiatric comorbidities: (1) “GTS+OCD” (excluding ADHD, but possibly other comorbidities), (2) “GTS+ADHD” (excluding OCD, but possibly other comorbidities), and (3) “GTS+OCD+ADHD” (and possibly other comorbidities). Patients not belonging to one of these subgroups were classified as “others.” Furthermore, a comorbidity score representing the individual's number of comorbid disorders (including OCD, ADHD, depression, and anxiety as defined above, range, 0–4) was calculated as suggested earlier (Freeman et al., 2000).

### Serum levels of aripiprazole

In order to investigate patient adherence, to quantify aripiprazole serum concentration and to correlate serum levels of aripiprazole with oral dosages of aripiprazole, we took 10 ml of blood for analysis of drug serum concentrations at follow-up.

### Statistical analyses

Statistical analysis was performed in the Statistical Package for Social Sciences (SPSS, Version 20.0 for Windows). Descriptive statistics (means, standard deviation, frequencies) were computed for all baseline characteristics. Associations between clinical assessments at baseline and follow up were examined via the Spearman rank correlation coefficient. In particular, correlates of PU, GTS-QoL, and GTS-QoL-VAS were computed. All statistical tests were two-sided and the alpha value was set at 0.05. No adjustment for multiple comparison (e.g., Bonferroni) was performed due to the exploratory nature of the analysis. Treatment effects in terms of pre/post-comparison of symptom severity of tics and comorbidities were carried out using the Wilcoxon-Mann-Whitney-Test for paired samples. The baseline characteristics of patients electing for- and against-treatment with aripiprazole were compared using the Wilcoxon-Mann-Whitney-Test for unpaired samples. Aripiprazole serum levels were correlated with administered oral dosage via Pearson correlation.

## Results

### Baseline characteristics

A total of 44 patients were included in the study [mean age = 39.4 (±12.2 (SD)] years, range, 18–58 years, female = 9, male = 35). Mean tic severity was 22.2 [(±8.5), range, 3–39] according to YGTSS-TTS and 9.6 [(±5.0), range, 0–18] according to MRVS. The sample exhibited a mean comorbidity score of 1.36 (range, 0–4) with a total of 9, 5, and 4 patients exhibiting 2, 3, and 4 comorbid conditions respectively. The diagnosis for comorbid OCD was made in 15 patients, for ADHD in 16, for depression in 14, and for anxiety in 15 patients. The subgroups of GTS depending on psychiatric comorbidities at baseline are shown in Table 1. Detailed clinical characteristics of the sample are presented in Table 2.

**Table 1:** **Subgroups of GTS depending on psychiatric comorbidities**.

**GTS Subgroups**	**Baseline**			**Follow-up**
	**Overall**	**Patients not treated with aripiprazole**	**Patients treated with aripiprazole**	
	***N* = 44**	***N* = 26**	***N* = 18**	***N* = 18**
GTS only	15	9	6	8
GTS+comorbidities	29	17	12	10
GTS+OCD	8	2	6	2
GTS+ADHD	8	8	1	1
GTS+OCD+ADHD	7	4	3	2
Others	6	3	2	5

*GTS, Gilles de la Tourette Syndrome; OCD, Obsessive-Compulsive Disorder; ADHD, Attention-Deficit/Hyperactivity Disorder; N, Number of cases; Others, Patients with comorbidities who does not fulfill criteria for one of the defined subgroups (comorbidities that fell into this category were depression N = 6, anxiety N = 6, depression+anxiety N = 4). Comorbidities include OCD, ADHD, depression, and anxiety (diagnoses as defined above)*.

**Table 2:** **Clinical characteristics at baseline and follow-up**.

	**Assessment**	**Patients not treated with aripiprazole (*N* = 26)**	**Patients treated with aripiprazole (*N* = 18)**		
		**Mean (SD) Baseline**	**Mean (SD) Baseline**	**Mean (SD) Follow-up**	**Difference between Baseline and Follow-up**
Tics	YGTSS -TTS	21.8 (±9.1)	22.7 (±7.9)	19.2 (+7.5)	−3.5^*^
	MT	12.8 (±4.5)	13.7 (±3.4)	11.8 (+3.4)	−1.9^*^
	VT	9.4 (±6.2)	9.0 (±5.8)	7.4 (±5.4)	−1.6^*^
	GS	42.5 (±17.8)	50.9 (±17.2)	35.9 (±17.4)	−15.0^**^
	MRVS	8.9 (±4.7)	10.4 (±5.5)	8.0 (±4.2)	−2.4^*^
PU	PUTS	21.8 (±5.9)	19.7 (±6.1)	19.8 (±5.5)	+0.1
OCD	Clinical diagnosis	*N* = 6	*N* = 9	*N* = 5	−4^*^
	M.I.N.I. OCD current	*N* = 6	*N* = 9	*N* = 7	−2
	Y-BOCS	3.7 (±6.9)	5.3 (±6.9)	4.2 (±5.5)	−1.1
	Obsessions	1.2 (±3.6)	1.7 (±3.5)	1.3 (±3.5)	−0.4
	Compulsions	2.5 (±4.4)	3.7 (±4.9)	3.0 (±4.9)	−0.7
	OCI-R	16.6 (±15.0)	15.5 (±11.6)	14.6 (±13.8)	−0.9
	Washing	0.9 (+1.9)	0.8 (±1.20)	1.6 (±2.85)	+0.8
	Obsessing	3.2 (±3.3)	2.7 (±2.6)	2.6 (±2.5)	−0.1
	Hoarding	3.2 (±2.7)	2.1 (±2.8)	2.1 (±2.9)	−
	Ordering	3.7 (±3.2)	4.3 (±3.3)	3.4 (±3.1)	−0.9
	Mental neutralization	1.4 (±2.1)	2.0 (±2.5)	2.2 (±2.9)	+0.2
	Checking	3.7 (±3.4)	3.6 (±3.3)	2.8 (±3.3)	−0.8
Depression	Clinical diagnosis	*N* = 8	*N* = 6	*N* = 4	−2
	M.I.N.I. MD current	*N* = 5	*N* = 3	*N* = 1	−2
	BDI-II	12.8 (±12.9)	13.7 (±10.8)	10.6 (±8.20)	−3.1
	MADRS	8.7 (±8.27)	7.2 (±5.6)	8.6 (±4.00)	+1.4
Anxiety	Clinical diagnosis	*N* = 9	*N* = 6	*N* = 4	−2
	M.I.N.I. panic current	*N* = 3	*N* = 1	*N* = 1	−
	agoraphobia	*N* = 4	*N* = 3	*N* = 3	−
	social phobia	*N* = 1	*N* = 0	*N* = 0	−
	GAD	*N* = 2	*N* = 1	*N* = 1	−
	BAI	13.8 (±13.8)	9.4 (±8.7)	8.6 (±7.0)	−0.8
Autistic traits	AQ	18.2 (±8.4)	19.3 (±7.7)	19.7 (±7.2)	+0.4
ADHD	Clinical diagnosis	*N* = 12	*N* = 4	*N* = 3	−1
	CAARS Inattention	50.4 (±13.2)	50.6 (±10.4)	47.9 (±7.3)	−2.7
	Hyperactivity-Restless	51.6 (±9.9)	51.9 (±11.3)	51.4 (±9.9)	−0.5
	Impulsivity	50.8 (±10.2)	52.5 (±11.9)	50.5 (±11.5)	−2
	Selfconcept	48.8 (±8.9)	47.6 (±8.9)	48.5 (±8.30)	+0.9
	Inattentive	53.1 (±15.6)	52.8 (±15.7)	54.7 (±13.9)	+1.9
	Hyperactive-Impulsive	51.4 (±13.3)	51.1 (±14.4)	51.5 (±9.4)	+0.4
	ADHD total	53.1 (±14.8)	52.3 (±14.9)	54.2 (±12.3)	+1.9
	ADHD index	53.1 (±11.2)	53.8 (±11.5)	53.8 (±9.6)	−
	WURS-k	35.3 (±15.5)	29.2 (±12.8)	−	−
	DSM-IV Attention	4.3 (±2.9)	4.3 (±3.2)	−	−
	Hyperactivity	3.5 (±3.1)	2.7 (±2.4)	−	−
QoL	GTS-QoL	33.4 (±23.8)	26.7 (±16.1)	24.5 (±17.1)	−2.2
	GTS-QoL-VAS	61.5 (±27.3)	60.6 (±20.5)	67.1 (±19.4)	+6.5

*YGTSS, Yale Global Tic Severity Scale; TTS, Total Tic Score; MT, Motor Tic Score; VT, Vocal Tic Score; GS, Global Score; MRVS, Modified Rush Video-Based Tic Scale; PU, Premonitory Urge; PUTS, Premonitory Urge for Tics Scale; OCD, Obsessive-Compulsive Disorder; M.I.N.I., International Neuropsychiatric Interview; Y-BOCS, Yale-Brown Obsessive Compulsive Scale; OCI-R, Obsessive-Compulsive Inventory Revised; MD, Major Depression; BDI, Beck Depression Inventory; MADRS, Montgomery Asberg Depression; GAD, Generalized Anxiety Disorder; BAI, Beck Anxiety Inventory, AQ, Autism-Spectrum-Quotient; ADHD, Attention-Deficit/Hyperactivity Disorder; CAARS, Conners Adult ADHD Rating Scale (for patients treated with aripiprazole, CAARS scores are given for N = 15/17 due to missing data), WURS-k, Wender Utah Rating Scale; DSM, Diagnostic and Statistical Manual; QoL, Quality of Life (the higher the sum, the lower the QoL); VAS, Visual Analogue Scale (the higher the sum, the higher the satisfaction). Clinical diagnosis, Diagnoses for comorbidities as defined above; N, Number of cases*.

* *p < 0.05*.

** *p < 0.01*.

Table 3 displays baseline correlates of PUTS, GTS-QoL, and GTS-QoL-VAS. PU (according to PUTS) did not correlate with tic severity [YGTSS-TTS (*r* = 0.281) and MRVS (*r* = 0.042)], but with Y-BOCS (*r* = 0.340), DSM-IV attention (*r* = 0.313), and CAARS ADHD total (*r* = 0.411).

**Table 3:** **Correlates of Premonitory Urges (as assessed by PUTS) and Quality of Life (as assessed by GTS-QoL and GTS-QoL-VAS) at baseline (*N* = 44)**.

**Assessments**	**PUTS**		**GTS-QoL**		**GTS-QoL-VAS**	
	***r***	***p*-value**	***r***	***p*-value**	***r***	***p*-value**
**TICS**						
YGTSS-TTS	0.281	0.065	0.461^**^	<0.01	−0.379^*^	0.011
YGTSS-GS	0.294	0.052	0.600^**^^a^	<0.01	−0.609^**^	<0.01
MRVS	0.042	0.790	0.139^a^	0.216	−0.330^*^	0.031
**OCD**						
M.I.N.I. OCD current	0.258	0.091	0.223	0.146	−0.316^*^	0.036
OCI-R	0.244	0.111	0.571^**^	<0.01	−0.509^**^^a^	<0.01
Y-BOCS	0.340^*^	0.024	0.277	0.069	−0.322^*^	0.033
**DEPRESSION**						
M.I.N.I. MD current	0.207	0.177	0.573^***^	<0.001	−0.589^***^	<0.001
BDI-II	0.276	0.070	0.776^**^	<0.01	−0.680^**^	<0.01
MADRS	0.293	0.054	0.790^**^	<0.01	−0.731^**^^a^	<0.01
**ANXIETY**						
M.I.N.I. panic	0.250	0.102	0.417^**^	0.005	−0.353^*^	0.019
M.I.N.I. agoraphobia	0.246	0.108	0.335^*^	0.026	−0.314^*^	0.038
M.I.N.I. social phobia	0.187	0.225	0.234	0.126	−0.253	0.098
M.I.N.I. GAD	−0.321^*^	0.034	0.185	0.230	−0.018	0.909
BAI	0.184^a^	0.229	0.672^**^	<0.01	−0.577^**^	<0.01
**ADHD**						
DSM-IV Attention	0.313^*^	0.038	0.360^*^	0.016	−0.217	0.157
DSM-IV Hyperactivity	0.110	0.478	0.073	0.636	0.082	0.595
CAARS ADHD total	0.411^*^	0.013	0.583^**^	<0.01	−0.302	0.073
WURS-k	0.256	0.098	0.421^**^	<0.01	−0.211	0.175
**AUTISTIC TRAITS**						
AQ	0.000	0.999	0.260	0.088	−0.209	0.174

*PUTS, Premonitory Urge for Tics Scale; GTS, Gilles de la Tourette Syndrome; QoL, Quality of Life (the higher the sum, the lower the QoL); VAS, Visual Analogue Scale (the higher the sum, the higher the satisfaction); YGTSS, Yale Global Tic Severity Scale; TTS, Total Tic Score; GS, Global Score; MRVS, Modified Rush Video-Based Tic Scale; OCD, Obsessive-Compulsive Disorder; M.I.N.I., International Neuropsychiatric Interview; OCI-R, Obsessive-Compulsive Inventory Revised; Y-BOCS, Yale-Brown Obsessive Compulsive Scale; MD,Major Depression; BDI, Beck Depression Inventory; MADRS, Montgomery Asberg Depression Scale; GAD, Generalized Anxiety Disorder; BAI, Beck Anxiety Inventory; ADHD, Attention-Deficit/Hyperactivity Disorder; DSM, Diagnostic and Statistical Manual; CAARS, Conners Adult ADHD Rating Scale; WURS-k, Wender Utah Rating Scale; AQ, Autism-Spectrum-Quotient. Correlation coefficient is given as r, Spearman correlation. N = Number of cases*.

* *p < 0.05*.

** *p < 0.01*.

*** *p < 0.001*.

*a= significant at follow-up*.

QoL (as assessed by GTS-QoL) correlated with a number of baseline characteristics significantly. We found the strongest correlations with assessments for depression [BDI-II (*r* = 0.776) and MADRS (*r* = 0.790)], followed by those for anxiety [BAI (*r* = 0.672)], ADHD [CAARS ADHD total (*r* = 0.583), WURS-k (*r* = 0.421), DSM-IV attention (*r* = 0.360)], and OCD [OCI-R (*r* = 0.571)]. Tic severity had only medium strength correlation [YGTSS-TTS (*r* = 0.461)]. Satisfaction-with-life (according to GTS-QoL-VAS) correlated with several baseline characteristics significantly: assessments for depression [BDI-II (*r* = −0.680) and MADRS (*r* = −0.731)], followed by those for anxiety [BAI (*r* = −0.577)] and OCD [Y-BOCS (*r* = −0.322) and OCI-R (*r* = −0.509)]. While YGTSS-TTS (*r* = −0.379) and MRVS (*r* = −0.330) had only weak correlations with GTS-QoL-VAS, YGTSS-GS correlated strongly with it (*r* = −0.609). There were no significant correlations with ADHD measures.

Autistic traits demonstrated no significant correlations with any of the above mentioned variables. Differences between males and females were not detected in any of the tests.

### Treatment effects of aripiprazole

18 of 44 patients elected for commencing treatment of their tics with aripiprazole. At follow-up, mean dosage of aripiprazole was 12.2 mg (median = 10 mg, range, 2.5–30 mg). All patients reported that they had reached their individual target dosage at the follow-up visit.

#### Tics and PU

Treatment with aripiprazole resulted in a significant tic reduction according to YGTSS (YGTSS-TTS: difference: −3.5, *p* = 0.027, YGTSS-MT: difference: −1.9, *p* = 0.037, YGTSS-VT: difference: −1.6, *p* = 0.045, YGTSS-GS: difference: −15.0, *p* = 0.002) and MRVS (difference: −2.4, *p* = 0.022) (see Table 2).

In contrast, aripiprazole did not result in a significant improvement of PU as assessed by PUTS (*p* = 0.917). Comparable to results at baseline, we observed no correlations between PU (according to PUTS) and tic severity (according to YGTSS and MRVS) for on-treatment patients. In contrast to results at baseline, at follow-up we only found a significant correlation between PU (according to PUTS) and BAI (*r* = 0.496, *p* = 0.036), but not with any other assessment for comorbidities.

#### Comorbidities

In relation to psychiatric comorbidities, our results indicated that aripiprazole caused a significant impact on psychiatric comorbidities. Specifically, the number of patients with the diagnosis of “GTS only” increased from *N* = 6 at baseline to *N* = 8 at follow-up, and the diagnosis of “GTS plus” decreased from *N* = 12 to *N* = 10 patients. With respect to above defined subgroups, the number of patients with “GTS+OCD” decreased from *N* = 6 to *N* = 2 patients, with “GTS+OCD+ADHD” from *N* = 3 to *N* = 2, but remained unchanged for the subgroup “GTS+ADHD” (*N* = 1) (see Table 1 for an overview).

**Figure 1:**
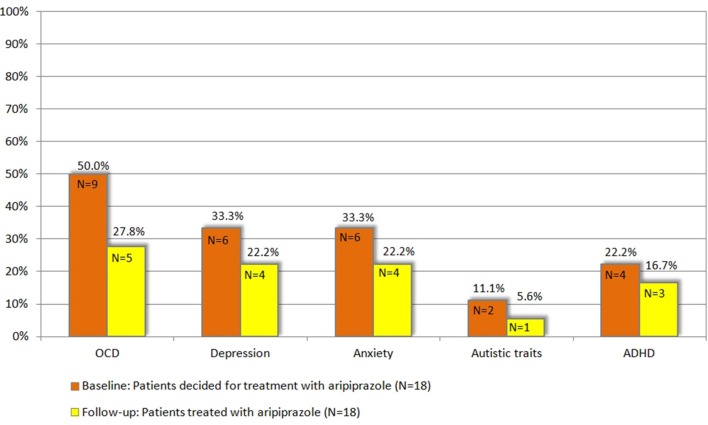
**Patients with respective comorbidity at baseline compared to follow-up, *N* = 18 (for definition of psychiatric comorbidities (OCD, ADHD, depression, anxiety, and autistic traits) refer to chapter 2.3)**.

With respect to the specific comorbidities, we discuss below all the clinical changes observed at follow-up in comparison to baseline (Figure 1). Treatment with aripiprazole resulted in a significant improvement of OCD. At baseline, the diagnosis of OCD was made in 9 patients (50%), but only in 5 patients (27.8%) after treatment (*p* = 0.046). None of the patients developed OCD during treatment. However, no significant changes were observed in the respective assessments for OCD at follow-up compared to baseline [Y-BOCS (*p* = 0.445), M.I.N.I. OCD current (*p* = 0.317), OCI-R (*p* = 0.585)].

For depression, although the total number of diagnosed patients decreased from 6 (33.33%) at baseline to 4 patients (22.2%) upon treatment, this decrease was not significant (*p* = 0.317). Notably, none of the patients developed comorbid depression while treated with aripiprazole. None of the different assessments for depression used in this study demonstrated a significant improvement [MADRS (*p* = 0.245), BDI-II (*p* = 0.201), M.I.N.I. MD current (*p* = 0.157)].

In 6 patients (33.33%) the diagnosis of anxiety disorder was made at baseline. After the treatment period, the diagnosis was still made in 4 patients (22.2%) resulting in a non-significant difference (*p* = 0.157). None of the patients developed an anxiety disorder during treatment with aripiprazole. Although BAI scores decreased in 12 patients, in none of the single tests a significant difference was seen [BAI (*p* = 0.163), M.I.N.I. social phobia/agoraphobia/panic current/GAD (*p* = 0.100)].

The diagnosis of comorbid ADHD was made in 4 patients (22.2%) at baseline and in 3 patients (16.7%) at follow-up (*p* = 0.271). However, we found no significant changes in respective assessments for ADHD at follow-up compared to baseline [CAARS ADHD total (*p* = 0.272)].

Accordingly, the mean comorbidity score decreased from 1.38 at baseline to 1.16 at follow-up. Specifically, at follow-up, 1 patient suffered from 1 comorbidity (compared to *N* = 5 at baseline), 8 patients from 2 (baseline *N* = 3), and 1 patient from 4 (baseline *N* = 2).

With respect to autism, of the 2 patients (11.1%), who exhibited pathologically autistic traits (according to AQ) at baseline, only 1 patient was still above the AQ cut-off at follow-up (*p* = 0.721). However, absolute AQ scores improved in 8 patients.

#### QoL and satisfaction-with-life

With respect to patient's QoL and satisfaction-with-life, treatment with aripiprazole resulted in a non-significant improvement as assessed by both GTS-QoL (difference: −2.2, *p* = 0.760) and GTS-QoL-VAS (difference: +6.5, *p* = 0.106). Comparable to baseline correlations in the whole sample (*N* = 44), we found significant positive correlations at follow-up (*N* = 18) between GTS-QoL and tic severity [YGTSS-GS (*r* = 0.581, *p* = 0.011) and MRVS (*r* = 0.616, *p* = 0.007)]. Once more, we found a negative and significant correlation between assessments for depression [MADRS (*r* = −0.663, *p* = 0.003)] and OCD [OCI-R (*r* = −0.492, *p* = 0.038)] with GTS-QoL-VAS. All other correlations with GTS-QoL and GTS-QoL-VAS were not significant.

Further details on clinical characteristics at follow-up (*N* = 18) are given in Table 2 and Figure 1. In none of the tests differences between male and female participants were detected.

#### Adverse effects and continuation of treatment

12 out of 18 patients (66.7%) reported AEs (for details see Table 4). However, the AEs experienced by the patients were not severe, as no medical intervention was necessary for any patient. After the end of the study, 14 patients (77.77%) decided to continue treatment with aripiprazole. Four patients stopped medication, among them three due to AEs [drowsiness, restlessness, sleep disturbance, restlessness of legs (akathisia)] and one due to no tic improvement. We found no differences between male and female participants with respect to reported AEs.

**Table 4:** **Reported adverse effects while taking aripiprazole (*N* = 18, multiple answers possible)**.

**Adverse effects**	***N* (%)**
Sleep disturbance	8 (44.4)
Restlessness	3 (16.7)
Restlessness of legs (akathisia)	1 (5.6)
Obstipation	2 (11.1)
Drowsiness	2 (11.1)
Hot flushes	1 (5.6)
Cardiac/chest pain	1 (5.6)
Weight gain	1 (5.6)
Feeling depressive	1 (5.6)

### Comparison of clinical characteristics of patients depending on their decision for/against treatment with aripiprazole

Of 44 patients included in this study, 18 patients elected to undergo treatment for their tics with aripiprazole [mean age = 38.5 (±13.7) years, range, 18–56 years, female = 4, male = 14] while 26 patients [mean age = 40 (±11.4) years, range, 18–58 years, female = 5, male = 21] elected for no treatment.

The reasons for the patients' decision were: (1) not interested in taking medication at all, (2) lack of disabling impairments by their tics, and (3) worries about possible AEs related to aripiprazole.

**Figure 2:**
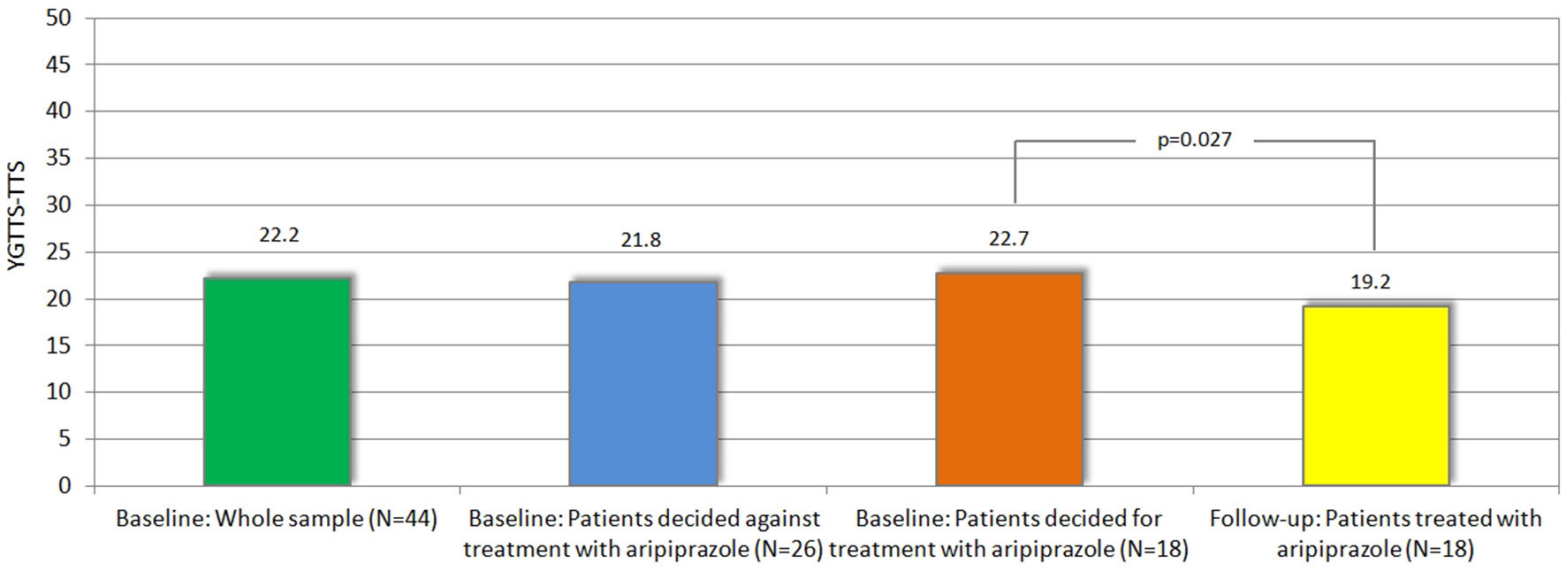
**Mean tic severity (according to YGTSS-TTS) at baseline (in whole sample and in those decided for vs. against treatment) and follow-up**.

**Figure 3:**
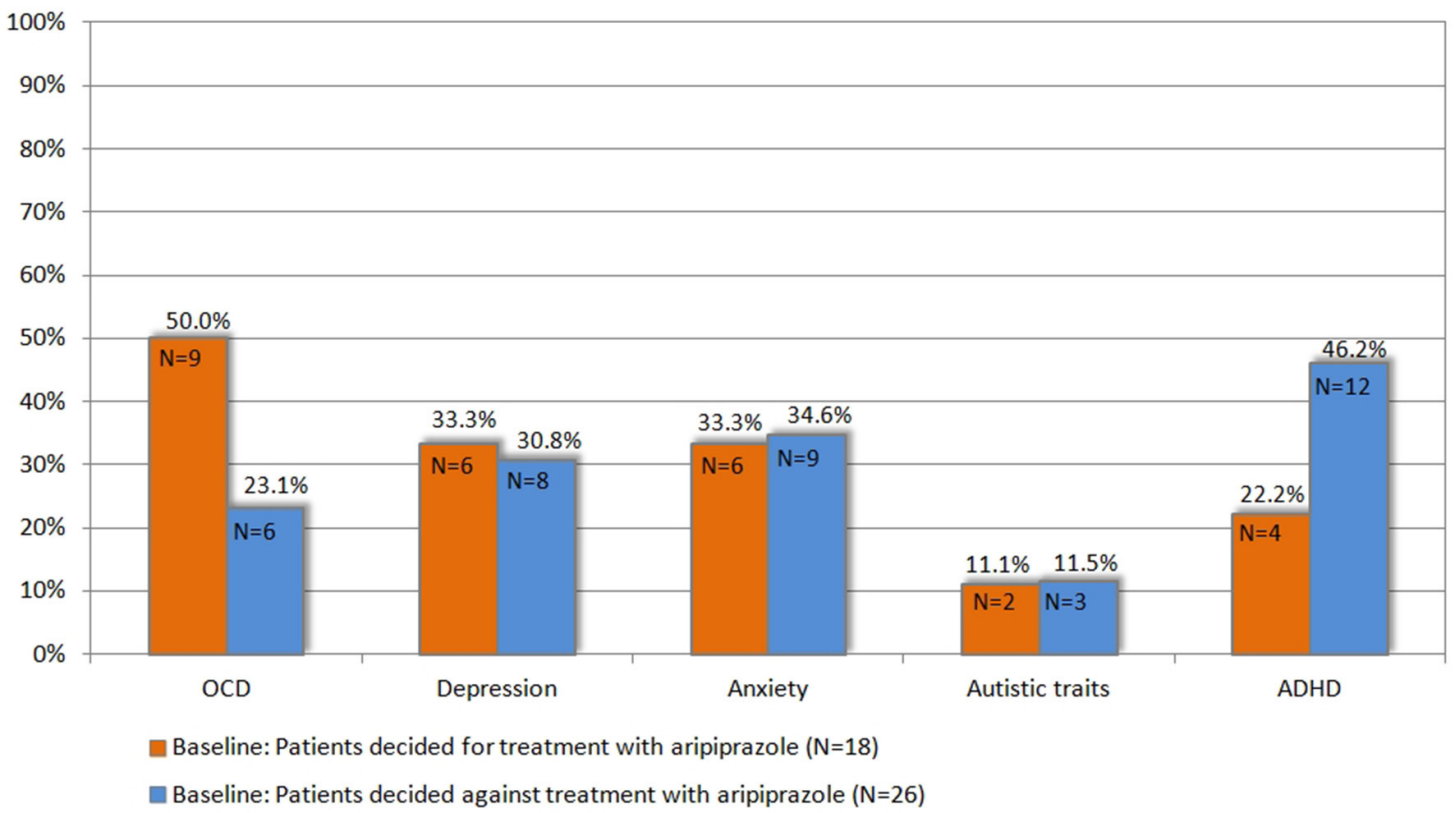
**Prevalence of comorbidities at baseline in patients who decided for vs. those who decided against treatment with aripiprazole (for definition of psychiatric comorbidities (OCD, ADHD, depression, anxiety, and autistic traits) refer to chapter 2.3)**.

Since this choice was solely based on the patients' own decision and neither on tic severity (according to YGTSS/MRVS; see Figure 2), nor the advice of the treating physician or the investigators, we compared the clinical characteristics between both groups at baseline in order to identify factors that may influence their decision for undergoing medical treatment for their tics. Most interestingly, neither tic severity (according to YGTSS and MRVS), nor PU (according to PUTS), nor QoL (as assessed by GTS-QoL and GTS-QoL-VAS) were significantly different between both groups. With respect to comorbidities (OCD, ADHD, depression, and anxiety), we observed that the diagnosis of comorbid OCD at baseline tended to be significantly more common in patients who decided for treatment with aripiprazole (9/18, 50%) compared to those who decided against the treatment (6/26, 23.1%, *p* = 0.067). With respect to comorbid ADHD, the opposite was the case as the diagnosis of ADHD was made less in patients who decided for treatment with aripiprazole (4/18, 22.2%) compared to those, who decided against (12/26, 46.2%, *p* = 0.109) (see also Figure 3).

When comparing both groups with respect to above defined subgroups, we found no significant differences. The diagnosis of “GTS only” was made in 6/18 (33.3%) patients, who decided for treatment with aripiprazole, compared to 9/26 (34.6%), who decided against treatment (*p* = 0.931). Accordingly, the diagnosis of “GTS plus” was made in 12/18 (66.7%) patients, who decided for treatment with aripiprazole, compared to 17/26 (65.4%) who decided against (*p* = 0.931) (subgroups: “GTS+OCD”: *N* = 2, “GTS+ADHD”: *N* = 8, “GTS+OCD+ADHD”: *N* = 4, others: *N* = 3) (see Table 1). Accordingly, the mean comorbidity score was comparable: 1.38 (range 0–4, *N* = 18) in patients, who decided for treatment and 1.34 (range 0–4, *N* = 26) in those, who decided against. When comparing results of distinct assessments for psychiatric comorbidities, no significant differences could be detected between both groups. Neither gender nor age had any influence on patients' decision for or against treatment.

### Serum levels of aripiprazole

Serum levels of aripiprazole were measured in 14/18 patients (missing blood samples in *N* = 4) ranging from 7.5 to 269 μg/L [mean = 125.3 (±79.8 (SD)] (therapeutic range: 150–250 μg/l). Serum levels correlated significantly with administered oral dosages of aripiprazole (2.5–30 mg/day) (*r* = 0.7, *p* = 0.003) indicating successful adherence to treatment.

## Discussion

This study is novel in two ways. Firstly, it is the first open-label clinical trial with a prospective investigation of the effect of aripiprazole on tic severity as well as on psychiatric comorbidities in adult patients with GTS. Secondly, our study design allowed us to investigate the factors that influence a patient's decision in undergoing pharmacotherapy of their tics for the first time. Our results indicated that (1) aripiprazole is effective and safe for the treatment of tics as well as for comorbid OCD and possibly other comorbidities such as ADHD, depression and anxiety; (2) aripiprazole has no influence on PU; (3) patients with comorbid OCD are more likely to elect for undergoing medical treatment for the treatment of their tics when compared to those with comorbid ADHD; and (4) neither tic severity, nor PU, nor QoL influence the patients decision making process.

### Efficacy of aripiprazole on tics and PU

Our findings are in line with preliminary data (Padala et al., 2005; Davies et al., 2006; Yoo et al., 2007, 2013; Budman et al., 2008; Lyon et al., 2009; Ghanizadeh, 2012; Wenzel et al., 2012) demonstrating that aripiprazole results in a significant improvement of tics in the majority of adult patients with GTS. This could be demonstrated by using both the examiner rating scale YGTSS-TTS and the video-based assessment MRVS.

In contrast, aripiprazole did not result in a significant improvement of PU. To the best of our knowledge, this is the first study, investigating the effect of aripiprazole on PU in adult patients with GTS. Thus, from our results it is suggested that effective treatment of tics is possible without improvement of PU. In addition, neither at baseline (*N* = 44), nor at follow-up (*N* = 18), significant correlations between PU (as measured by PUTS) and tic severity (according to YGTSS-TTS and -GS and MRVS, respectively) were found. These findings, therefore, further support recent clinical studies suggesting that PU is not as closely related to tic severity as previously assumed (Ganos et al., 2012; Müller-Vahl et al., 2014). In line with these findings, from an increasing number of brain imaging studies it is suggested that different brain areas are involved in the occurrence of PU (supplementary motor area, insula, and mid-cingulate cortex) and tics (thalamus, central operculum, primary motor, somatosensory, premotor, and parietal cortices) (Bohlhalter et al., 2006; Jackson et al., 2011; Neuner et al., 2014).

Our results showed a positive correlation between PU (according to PUTS) and anxiety (according to BAI) at baseline. This finding is in line with recent data demonstrating on the one hand stronger PU in GTS patients with comorbidities compared to those without and on the other hand a positive correlation between PU and OCD (Eddy and Cavanna, 2013; Sambrani et al., 2016).

### Diagnoses for comorbidities

For a thorough clinical characterization of our sample, a number of clinical assessment tools were utilized to diagnose and quantify each of the commonly associated psychiatric comorbidities. Specifically, 3 rating scales were used for each of obsessive-compulsive symptoms inattention/hyperactivity and depressive symptoms, while 2 rating scales were used for anxiety.

Nevertheless, it is well known that the use of different assessments—although developed for the measurement of the same symptoms—may reflect different sides of the same disorder and therefore, may lead to inconsistent findings. In addition, it is generally accepted that patients' self-perception may differ from professional evaluation (Beblo and Lautenbacher, 2015) resulting in discrepant results when using self-ratings compared to examiner rating scales (Olariu et al., 2015). For example, the subtle differences of commonly used depression rating scales were pointed out by Uher et al. (2008), who showed that the Hamilton Rating Scale for Depression (HAMD-17), MADRS and BDI-II, while all being valid and reliable scales, reflect “internally consistent but mutually distinct estimates of depression severity.” While the MADRS is closer to the core of depression—observable from the outside—the BDI represents a “cognitive” dimension—which is more of an internal experience. Uher et al. (2008), therefore, recommended to use these scales in a complementary fashion. Comparably, we found contrary results when using the BDI-II as a self-rating compared to the MADRS as an expert rating: while treatment with aripiprazole resulted in a decrease in mean BDI-II values, mean MADRS scores increased. A closer investigation showed that this obvious inconsistency was due to the high incidence of “sleep disturbances” (44.4%) and “internal unrest” (16.7%), which were the most frequently reported AEs during treatment with aripiprazole. While having little impact on the BDI-II score, the presence of these symptoms had a considerably high impact on the MADRS scores leading erroneously to high scores for depression.

Similar findings were reported for OCD, where the Y-BOCS was shown to represent a better measure for symptom severity and the OCI-R was shown to be primarily a measure of symptom presence (Sulkowski et al., 2008) and for ADHD ratings (Rösler et al., 2006). Taylor et al. (2011) report CAARS and WURS-k as the most robust ADHD scales with content validity, compared with 12 other ADHD scales. The same was true for anxiety ratings, where the discriminant validity of generally used anxiety screening was reported (Phan et al., 2016). In addition, limitations of the M.I.N.I. in assigning anxiety disorders are well known, since it includes only four, but not all categories for anxiety disorders as defined in the ICD-10.

To overcome these methodological difficulties and to help balance the advantages and disadvantages of self-report and clinician-ratings, we therefore decided to make the diagnoses of both OCD, ADHD, depression and anxiety, not only based on any single (arbitrarily selected) rating scale, but on the combination of several scales. While this procedure is well accepted for the diagnosis of ADHD (Rösler et al., 2006), it is less established in the context of other diagnoses such as OCD, depression, and anxiety. We are aware that in most other clinical trials diagnoses of comorbidities and changes during treatment are only based on one single assessment. However, we believe that this concept may result in more robust diagnoses and may avoid false positive and negative results. In addition, we want to stimulate a discussion about the diagnostic procedure of comorbidities in patients with GTS. It is well known that both depressive (Trillini and Müller-Vahl, 2015b) and OCD symptoms (Worbe et al., 2010) in this group of patients may differ from those in patients without GTS and therefore well-established instruments for these diagnoses (without GTS) might be less suitable in patients with GTS.

### Efficacy of aripiprazole on comorbidities

According to diagnoses defined in these terms, treatment with aripiprazole resulted in a significant improvement of OCD (although respective assessments for OCD demonstrated no significant changes at follow-up compared to baseline). Due to the small number of patients, results in other psychiatric comorbidities did not reach statistical significance. However, according to this concept, treatment with aripiprazole, in addition, resulted in remission of comorbid depression in 4 of 6 patients, of comorbid anxiety in 4 of 6 patients, and of comorbid ADHD in 1 of 4 patients. Accordingly, the mean comorbidity score decreased from 1.38 at baseline to 1.16 at follow-up. Furthermore, pathologically autistic traits changed in 1 of 2 patients and absolute scores (according to AQ) improved in 8 patients.

Our findings corroborate available case reports in patients with GTS reporting about beneficial effects of aripiprazole in the treatment of depression (Murphy et al., 2009; Wenzel et al., 2012), OCD (Murphy et al., 2005, 2009; Winter et al., 2008), anxiety, self-injurious behavior (Wenzel et al., 2012), inattention (Murphy et al., 2009), and ADHD (Masi et al., 2012), but in contrast to preliminary results by Frölich et al. (2010) who found no improvement of ADHD and OCD in children with GTS. However, in none of these studies the effect of aripiprazole has been investigated specifically for the treatment of psychiatric comorbidities, comorbidities were not assessed by using a variety of self- and examiner-ratings, and, at least in part, data were collected retrospectively from patient records.

Furthermore, our findings in patients with GTS are completely in line with data in patients with pure OCD, where aripiprazole has been found to be effective not only in the treatment of uncomplicated OCD (Sayyah et al., 2012), but even in patients resistant to treatment with selective serotonin reuptake inhibitors (SSRI) (Delle Chiaie et al., 2011; Masi et al., 2013; Dold et al., 2015; Shoja Shafti and Kaviani, 2015). Aripiprazole has also been found helpful in the treatment of depression (Wen et al., 2014) and anxiety disorders (Pae et al., 2008; Katzman, 2011). It is even one of the most often prescribed medication in patients with anxiety and mood disorders (Carton et al., 2015). Although often prescribed in ADHD (Carton et al., 2015) its efficacy has not been shown (Ghanizadeh, 2013).

The effective influence of aripiprazole on a number of psychiatric conditions has been suggested to be a result of its unique pharmacological profile. Specifically, aripiprazole is a functionally selective drug that exhibits an adaptive pharmacological profile that is dependent on the local levels of the endogenous ligands. Aripiprazole is a partial dopamine D2 agonist, a partial serotonin 5-HT1A agonist, and a 5-HT2A antagonist. Apart from its recognized influence on the dopaminergic and serotonergic systems, aripiprazole has also been shown to modulate the glutamatergic and GABAergic neurotransmitter systems (De Bartolomeis et al., 2015). Therefore, it can be speculated that beneficial effects of aripiprazole on OCD, depression and anxiety in patients with GTS may be the result of its ability to selectively and adaptively stabilize multiple neurotransmitter systems.

### Influence of aripiprazole on quality of life

Comparable to previous reports by Müller-Vahl et al. (2010) and Jalenques et al. (2012), we found that in adult patients with GTS both QoL and satisfaction-with-life (as assessed by GTS-QoL and GTS-QoL-VAS) are mainly impaired by depression. This was true at baseline and also during treatment with aripiprazole. Although aripiprazole resulted in a significant improvement of tics, we only found a trend toward an improvement in patients' QoL. This is in line with the finding that depression influences patients' QoL more than the tics. Interestingly, we found a significant correlation between PU and QoL at baseline. Assuming that PU is a kind of an OCB as suggested recently (Sambrani et al., 2016) and against the background that it is well-known that OCD significantly impairs QoL in adult patients with GTS (Müller-Vahl et al., 2010), this correlation can possibly be explained by the negative influence of OCD on patients' QoL. The complex interplay between tics, comorbidities, and QoL is also expressed in changes in the YGTSS-GS: this “global score” of the YGTSS is a measurement for both tic severity and overall impairment. Completely in line with all above mentioned results, after treatment with aripiprazole we found a much greater reduction of the YGTSS-GS (*p* = 0.002) compared to the tic score of the YGTSS (YGTSS-TTS, *p* = 0.027).

### Adverse effects of aripiprazole

Although AEs were reported by a substantial number of patients (66.7%), most AEs were mild and/or tolerable corroborating recent data that in most adult patients with GTS aripiprazole is well tolerated (Lyon et al., 2009; Wenzel et al., 2012; Diomšina et al., 2015). No serious AEs occurred. Three of eighteen patients (16.7%) decided to stop treatment with aripiprazole due to AEs such as drowsiness, restlessness, sleep disturbance, and restlessness of legs (akathisia). Comparable to our data, Wenzel et al. (2012) also reported about the occurrence of AEs in nearly 2/3 (59%) of their patients. However, in contrast to our results, they found drowsiness (20%) to be the most common side effect, while sleep disturbances were quite rare (9%). In this study, sleep disturbances (44.4%) followed by restlessness (16.7%) were the most often reported AEs, while drowsiness occurred in only 11.1% of our patients. This difference might be explained by different study designs and different treatment durations (4–6 weeks in our study vs. 1–60 months in Wenzel et al., 2012). Hence, it can be assumed that in the context of a longer-term treatment, patients may tolerate drowsiness rather than restlessness and sleep disturbances. Nonetheless, our data further support the clinical practice to start treatment with aripiprazole once daily in the morning, and to postpone intake to the evening, if significant drowsiness occurs.

### Decision factors for treatment with aripiprazole

Our study design provided us with the possibility of investigating the factors that influence patient's decision in electing for—or against—treatment. This choice was solely based on each patient's own preference. We found no differences between both groups with respect to age, gender, and comorbidity score. Most interestingly, neither tic severity (according to YGTSS and MRVS), nor PU (according to PUTS) nor QoL (as assessed by GTS-QoL and GTS-QoL-VAS) was significantly different between both groups. However, we found a trend with respect to comorbid OCD and ADHD. While OCD was more common in those patients who decided for treatment with aripiprazole, ADHD was more common in those who decided against. Thus, although not reaching the significance threshold, our data seems to indicate that there are aspects influencing patients' decisions for or against medical treatment for tics beyond tic severity. Since comorbid OCD has a strong negative impact on patients' QoL, it can be speculated that this might be a driving force that also influences patients' treatment decision in favor of treatment for tics. However, it can also be possible that patients with comorbid OCD differ in their assessment of impairment caused by their tics as compared to patients without OCD, possibly due to a larger extent of ruminating and worrying caused by their compulsions. Finally, it can be hypothesized that patients with comorbid ADHD are less impaired by their tics and therefore tend to decide against treatment. This is particularly noteworthy, since it has been demonstrated that patients with comorbid ADHD are less able to suppress their tics (Sambrani et al., 2016) and effective tic suppression has a positive impact on patients' QoL (Matsuda et al., 2016).

### Characteristics of the sample and serum levels of aripiprazole

With respect to tic severity, comorbidities, and distribution of gender, in this open-label study a representative clinic sample of adult patients with GTS was included. Although, all patients participating in this study, in addition, participated in an MRI study—and therefore patients also had to fulfill inclusion criteria for that study—our group of patients was characterized by moderate tics (mean tic severity = 22.2 according to YGTSS-TTS, *N* = 44). Usually, a threshold of YGTSS-TTS >14 indicates clinically significant tics that justify treatment (Leckman et al., 1989; Wilhelm et al., 2012). In contrast to most other studies investigating the efficacy of aripiprazole in patients with GTS, we included only adults > age of 18 years. It is noteworthy that all patients who received treatment with aripiprazole were otherwise free of any other psychoactive drug for at least 4 weeks before entering the study. Thus, interactions with other psychoactive substances or augmentation effects are not of concern. Measurements of serum levels of aripiprazole demonstrated patient adherence. For the first time, we were able to show positive correlation between oral dosage and serum levels of aripiprazole in this group of patients.

### Limitations

There are the following limitations of the study: (1) no patient control group with either placebo or another active drug was included, (2) we included different groups of patients (mildly vs. severely affected patients, pretreated vs. drug-naive patients, and patients with vs. without comorbidities), (3) the number of patients undergoing treatment was relatively low, (4) given that treatment duration was relatively short (only 4–6 weeks) and that (5) aripiprazole's long half-life of approximately 72 h, we cannot rule out that aripiprazole levels were still increasing at the follow-up assessment. (6) Most of the patients were recruited from the Clinic of Psychiatry, Socialpsychiatry and Psychotherapy at the MHH and, therefore, we cannot exclude a bias toward more severely and complex affected patients as well as a selection bias, (7) only those patients were assessed who agreed to participate in the study and only those were reassessed who decided in favor of treatment with aripiprazole, which decreases the external validity of our trial, (8) we cannot exclude that patients decided for participation in the study at baseline and/or follow-up due to monetary compensation, and (9) declining the participation at follow-up due to reluctance of travel and/or discomfort in the MRI, (10) from our data, it cannot be excluded that other factors than comorbid OCD and ADHD may influence patients' decisions making process for or against medical treatment of their tics.

The strengths of this study are: (1) the inclusion of a relatively large number of patients, (2) at baseline, all patients were drug-free, (3) a combination of several validated assessment instruments insured the integration of the benefits of both clinician rating and self-ratings for the diagnoses of psychiatric comorbidities including OCD, ADHD, depression and anxiety, (4) monotherapy with aripiprazole, enabled the exclusion of influence of interactions with other drugs, (5) direct comparison of groups of patients, electing for and against treatment with aripiprazole was conducted, (6) confirmation of patients' treatment adherence by determination of serum levels of aripiprazole, and (7) the self-selection of patients in our study has a high external validity, since these would be the patients opting for aripiprazole in the clinic.

## Conclusion

To the best of our knowledge, this is the first prospective open-label clinical trial with a larger sample examining untreated adult patients with GTS before and after a treatment period of 4–6 weeks with aripiprazole monotherapy. The major findings of the study are: (1) aripiprazole results in significant reduction of tics in adult patients with GTS, but it does not affect PU; (2) aripiprazole results in significant reduction of OCD and possibly other comorbidities including depression, anxiety, and ADHD; (3) patients with GTS with comorbid OCD tend to decide for treatment of tics with aripiprazole, whereas patients with comorbid ADHD tend to decide against this kind of medication; (4) neither tic severity, nor PU or QoL influence patients' decision making process for or against treatment of their tics with aripiprazole, (5) aripiprazole appears safe and AEs are commonly tolerable; and (6) patients' QoL is mostly impaired by comorbid depression. For further clinical trials it is suggested to use a large variety of different rating scales in order to capture and assess psychiatric comorbidities in patients with GTS.

## Author contributions

SG: Acquired the clinical data, Performed the analysis, Wrote the paper. AK: Acquired the MRI data, Contributed to the writing of the manuscript. EJ: Contributed to the writing of the manuscript. KM: Wrote the paper.

### Conflict of interest statement

The authors declare that the research was conducted in the absence of any commercial or financial relationships that could be construed as a potential conflict of interest. The reviewer DR and handling Editor declared their shared affiliation, and the handling Editor states that the process nevertheless met the standards of a fair and objective review.
